# A Glutamic Acid-Producing Lactic Acid Bacteria Isolated from Malaysian Fermented Foods

**DOI:** 10.3390/ijms13055482

**Published:** 2012-05-07

**Authors:** Mohsen Zareian, Afshin Ebrahimpour, Fatimah Abu Bakar, Abdul Karim Sabo Mohamed, Bita Forghani, Mohd Safuan B. Ab-Kadir, Nazamid Saari

**Affiliations:** Faculty of Food Science and Technology, Universiti Putra Malaysia, Serdang 43400, Selangor, Malaysia; E-Mails: mzar89@yahoo.com (M.Z.); a_ebrahimpour@yahoo.com (A.E.); fatim@putra.upm.edu.my (F.A.B.); ak@food.upm.edu.my (A.K.S.M); arezoebita@yahoo.com (B.F.); safuankadir1207@gmail.com (M.S.B.A.-K.)

**Keywords:** γ-amino butyric acid, *Lactobacillus plantarum*, 16S rRNA gene sequencing, polymerase chain reaction, phenotypic identification, sugar assimilation profile

## Abstract

l-glutamaic acid is the principal excitatory neurotransmitter in the brain and an important intermediate in metabolism. In the present study, lactic acid bacteria (218) were isolated from six different fermented foods as potent sources of glutamic acid producers. The presumptive bacteria were tested for their ability to synthesize glutamic acid. Out of the 35 strains showing this capability, strain MNZ was determined as the highest glutamic-acid producer. Identification tests including 16S rRNA gene sequencing and sugar assimilation ability identified the strain MNZ as *Lactobacillus plantarum.* The characteristics of this microorganism related to its glutamic acid-producing ability, growth rate, glucose consumption and pH profile were studied. Results revealed that glutamic acid was formed inside the cell and excreted into the extracellular medium. Glutamic acid production was found to be growth-associated and glucose significantly enhanced glutamic acid production (1.032 mmol/L) compared to other carbon sources. A concentration of 0.7% ammonium nitrate as a nitrogen source effectively enhanced glutamic acid production. To the best of our knowledge this is the first report of glutamic acid production by lactic acid bacteria. The results of this study can be further applied for developing functional foods enriched in glutamic acid and subsequently γ-amino butyric acid (GABA) as a bioactive compound.

## 1. Introduction

Glutamic acid is a multifunctional amino acid involved in taste perception, excitatory neurotransmission and intermediary metabolism [1]. It plays an important role in gastric phase digestion with multiplicity effects in the gastrointestinal tract when consumed with nutrients by enhancing gastric exocrine secretion [2]. Glutamic acid is a specific precursor for other amino acids *i.e.*, arginine and proline as well as for bioactive molecules such as γ-amino butyric acid (GABA) and glutathione. GABA possesses several well-known physiological functions (*i.e.*, anti-hypertension [3] and anti-diabetic [4]) and glutathione plays a key role in the protection of the mucosa from peroxide damage and from dietary toxins [5]. Furthermore, a number of studies have shown the possible usefulness of glutamic acid in enhancing nourishment in the elderly and in patients with poor nutrition [6,7]. At the present time, glutamic acid is largely produced through microbial fermentation because the chemical method produces a racemic mixture of glutamic acid (d- and l-glutamic acid) [8]. Numerous studies have reported glutamic acid excretion by various micro-organisms; however, most of them were not food-grade micro-organisms. Lactic acid bacteria (LAB) are well known to produce a variety of primary metabolites. The existence of the *gdh* gene in LAB which is responsible for the production of glutamic acid has been proven [9]. In addition, LAB are essential in the processing of food materials and they have been applied extensively in the food industry [10]. LAB can enhance the shelf-life and safety of foods, improve food texture, and contribute to the nutritional value of food products and pleasant sensory profile of the end-use products [11]. Employing LAB, on the other hand, with the potential to produce glutamic acid can facilitate production of functional foods rich in bioactive molecules such as GABA. The key advantage of the glutamic acid production by LAB is that the amino acid produced in this way is biologically active (l-glutamic acid) and the production process is considered to be safe and eco-friendly. This can be achieved through selection of appropriate LAB strains from indigenous micro-organisms which are well-adapted to a particular product, more competitive and with elevated metabolic capacities. Thus, research of this technological potential is of highest interest to the industry.

As a result, selection of the most efficient glutamic acid-producing LAB strains may contribute to new fermented products with improved general standards with respect to the naturally-biosynthesized glutamic acid. This could lead to the development of fermented foods rich in glutamic acid and consequently GABA. Therefore, it is hypothesized that screening various LAB capable of producing glutamic acid may explore new avenues for mass production of functional foods rich in GABA.

## 2. Results and Discussion

In this study, a number of indigenous fermented foods available in Malaysia were used for the isolation of lactic acid bacteria. These isolated LAB strains were individually examined based on their potential to produce glutamic acid. Table 1 summarizes the characteristics of such LAB exhibiting the capacity to produce glutamic acid. It shows that 14 strains of 35 that produced glutamic acid were isolated from fermented soybean. Higher concentrations of glutamic acid produced by LAB strains isolated from fermented soybean showed that these strains were more efficient in biosynthesizing glutamic acid as compared to LAB strains isolated from other food samples. Among fermented food-derived LAB strains, only one strain showed a superior glutamic acid-producing potential with a contribution of 489.46 μmol/L glutamic acid. This strain assigned as MNZ, was subjected to further phenotypic and genotypic identification tests.

Glutamic acid production was previously reported by Zalán *et al*. (2010) [12] and Tarek and Mostafa (2009) [13] for some of the LAB species such as *Lactobacillus paracasei* and *Lactobacillus* spp.; however, in this study, *Lactobacillus plantarum* was reported as a glutamic acid producer. The level of glutamic acid produced by LAB in other studies was reported to be 68.7 mg/L [13] and <25 mmol/L [12]. Gram-positive micro-organisms other than LAB were also shown to produce glutamic acid. For example, *Brevibacterium* spp. were found to produce this amino acid between 10 to 46 mmol/L [14].

### 2.1. Phenotypic and Genotypic Identification

In order to identify the LAB strain MNZ, the API carbohydrate fermentation kit was utilized. The results showed that this strain was able to assimilate the following carbon sources: Ribose, galactose, d-glucose, fructose, mannose, manitol, sorbitol, *α*-methyl-d-mannoside, *N*-acetyl glucosamine, arbutin, cellobiose, maltose, lactose, melibiose, sucrose, gentiobiose, turanose, salicin and aesculin; while this ability was negative for the rest of carbon sources. The pattern of assimilation ability of this isolate for carbohydrates was in agreement with the metabolic characteristics of *Lactobacillus plantarum* described by Bergey’s Manual of Systemic Bacteriology [15]. Taxonomic identification of the strain MNZ at species level was performed by 16S rRNA gene sequencing. The sequence was deposited on NCBI under accession number HM175883 (*Lactobacillus plantarum* MZ-02).

### 2.2. Effect of Various Carbon Sources on Glutamic Acid Production

**Figure 1:**
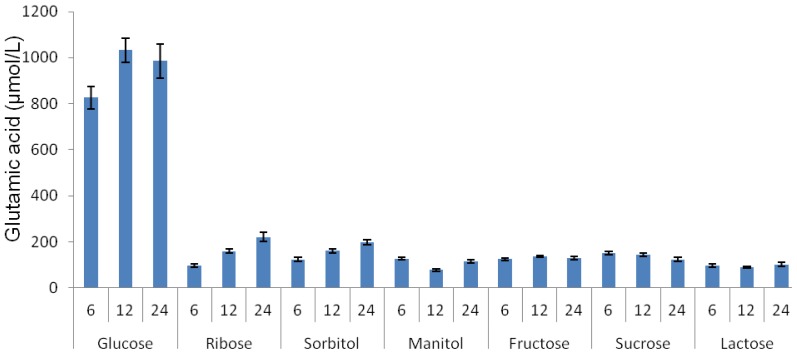
Effect of different carbon sources with different concentrations on the production of glutamic acid by the LAB strain MNZ cultured in MRS medium at 30 °C for 120 h. Values are mean ± standard deviation of two independent experiments.

The effect of different carbon sources on the production of glutamic acid by the LAB strain MNZ was investigated in this study. It was found that the following carbohydrates were fermented by *Lactobacillus plantarum* MNZ: Ribose, sorbitol, manitol, fructose, glucose, sucrose and lactose. Three concentrations (6, 12 and 24% *w*/*v*) were tested for each carbon source and the production of glutamic acid was determined in MRS medium supplemented with each carbon source individually at 30 °C for 120 h. Based on a preliminary test (data not shown), the range of 6–24% (*w*/*v*) was found to be the most suitable for assessment of various carbon sources. The results presented in Figure 1 indicate that glucose was the best source of carbohydrate for the production of glutamic acid among the tested sources where 12% was found to be the optimum point for glutamic acid production. Since 12% and 24% glucose concentrations did not show any significant differences (*p* < 0.05) regarding the production of glutamic acid by the LAB strain MNZ, the lowest amount was chosen for further studies

In the present study, a number of various carbon sources was tested to select the most suitable one for the purpose of glutamic acid production by the LAB strain MNZ. Media containing various carbon sources have already been used for the production of amino acids by the bacteria [16]. In case of the microbial glutamic acid production, a number of different carbon sources such as glucose, sucrose, fructose, ribose, *etc*. have been tested and similar results were reported compared to the results obtained in this study. For example, Roy and Chatterjee [17] reported that fructose and glucose in order of effectiveness were the best carbon sources for the glutamic acid production in a synthetic media. Other carbon sources including sucrose and manitol showed far less effect on glutamic acid production. The best glucose concentration (8%) reported by Roy and Chatterjee [17] was lower than the one found in this study (12%).

This study demonstrates the effectiveness of glucose as an appropriate carbon source in glutamic acid production compared to other sugars, especially those that are not hexoses, such as ribose, lactose and sucrose. The higher growth rate of lactic acid bacteria on glucose as a hexose sugar compared to non-hexoses [18] could be the reason for such an elevated glutamic acid production from glucose. On the other hand, glutamic acid was found to be produced in bacteria from glucose via Krebs cycle intermediates [19]. The carbon-energy source of glucose can be converted into pyruvic acid by the glycolysis pathway, a preface to the TCA cycle and the electron-transport chain [20]. Therefore, glucose was found to be the most appropriate sugar for glutamic acid production by the LAB strain MNZ in this study.

The result of the present study concerning the effects of different carbon sources on glutamic acid biosynthesis revealed that optimum levels of glucose can effectively promote glutamic acid production possibly through the Krebs cycle. Such findings can be further used for appropriate selection of other substrates containing glucose at higher levels for enhanced production of glutamic acid.

### 2.3. Study of Glucose Consumption

**Figure 2:**
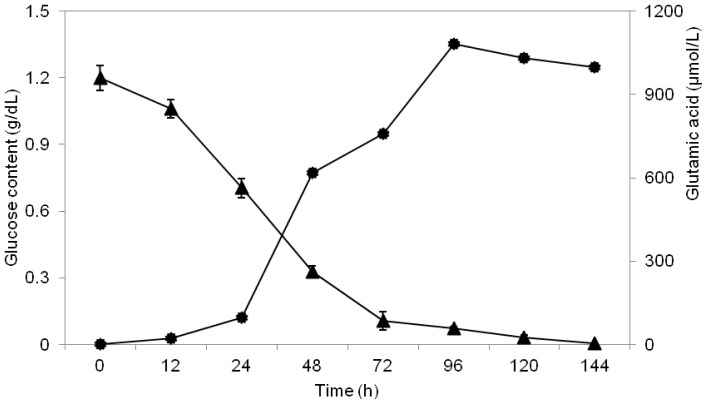
Consumption of glucose (g/dL) by the LAB strain MNZ cultured in MRS medium supplemented with 12% glucose at 30 °C. Symbols used were: (▴), Glucose consumption profile of the bacterium; (●), glutamic acid production.

In order to investigate the glucose consumption pattern of the LAB strain, the strain MNZ was cultivated in MRS broth at 30 °C for 144 h. The broth was supplemented with 12% glucose in order to examine the effects of glucose concentration on the production of glutamic acid. As shown in Figure 2, there was a sharp decrease in glucose content between 12 and 72 h of fermentation. This might be due to the microbial growth with the highest log_10_ CFU/mL of 8.3 after 72 h of fermentation. In other investigations using other species of glutamic acid-producing bacteria, the glucose consumption pattern during glutamic acid production was found to be similar to the present study [21]. A continuing decrease in the sugar concentration which is consumed by the bacterium corresponds with the growth of the culture [22].

### 2.4. Effect of Different Concentrations of Ammonium Nitrate on Glutamic Acid Production

**Figure 3:**
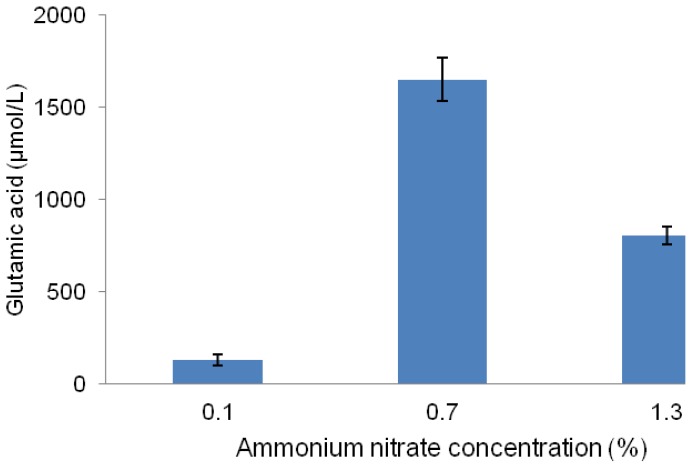
Effect of different concentrations of ammonium nitrate on glutamic acid production by the LAB strain MNZ cultured at 30 °C in MRS.

Ammonium nitrate as a nitrogen source was chosen in this study and the effect of its different concentrations on the glutamic acid production was tested. The results presented in Figure 3 show that 0.7% (*w*/*v*) was the best concentration of ammonium nitrate for *Lactobacillus plantarum* to produce glutamic acid. The mechanism by which ammonia enhances glutamic acid production is associated with nitrogen as an essential component for amino acid production. Nitrogen plays an important role in fermentative cultivation of glutamic acid-producing bacteria. Therefore, nitrogen is taken up by cells, and thereafter assimilated to accomplish their metabolism [23].

It is well-known that the uptake of nitrogen sources into the cells is mediated either by passive diffusion (ammonium) or active transport [24]. Generally the bacteria follow two different pathways for ammonium assimilation to form glutamic acid. When the ammonia concentration is low and the diffusion into cells becomes restricted, a unique ammonium transporter (AmtB) encoded by the *amt* gene is activated to cope with the nitrogen starvation, and ammonium is assimilated to glutamine by glutamine synthetase [25]. In contrast, in the presence of high concentrations of ammonium, the diffusion of uncharged ammonia (NH_3_) occurs through the cytoplasmic membrane. This would promote the growth of the bacterial cells, and thereby ammonium is assimilated by glutamate dehydrogenase to form glutamic acid. Similarly, Tesch *et al*. [26] showed that most of the ammonium (72%) is assimilated by the glutamate dehydrogenase to form glutamic acid which has been proven to demonstrate high activity in *Lactobacillus plantarum* [27].

### 2.5. pH Association with Glutamic Acid Production

**Figure 4:**
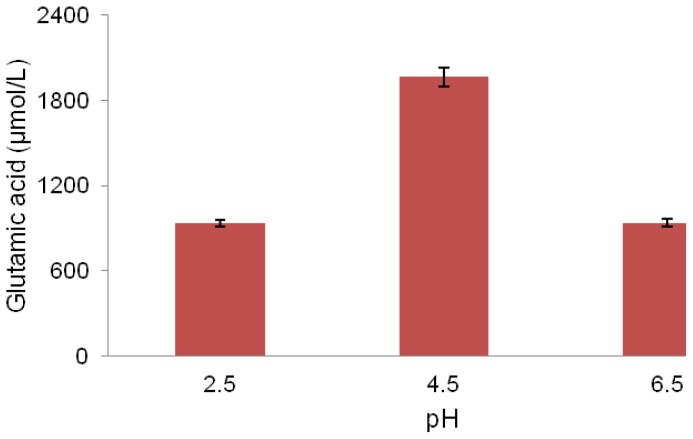
Effect of initial pH on glutamic acid production by the LAB strain MNZ cultured at 30 °C in MRS medium.

Effect of various initial pH on glutamic acid production by the LAB strain MNZ was investigated in this study. Results show that an initial pH value of 4.5 was the best compared to other pH values (Figure 4). pH plays an important role in biological processes and the pH of the medium is important for the glutamic acid production [28]. *Lactobacillus plantarum* prefers a moderately acidic pH 6.5 for optimal growth [29]. However, it was noted that the maximum glutamic acid production in this study occurred at a lower pH (4.5). The pH of the culture medium can influence the growth rate of *Lactobacilli* [29]. An initial pH value of lower than 6.5 decreases the growth rate of *Lactobacillus plantarum* in the medium [29]. According to Krämer [30], glutamic acid secretion occurs by an overflow metabolism whenever growth is limited. This could cause a redirection of 2-oxoglutarate efflux towards glutamic acid production which leads to an increase of the glutamic acid excretion rate [31].

It was also proved that *Lactobacillus plantarum* produces ammonia in an acidic environment, which contributes to pH homeostasis and thereby survival of the micro-organism through neutralizing the pH [32]. As a result, the ammonia produced this way can be utilized in glutamic acid production. Thus, the ability of *Lactobacillus plantarum* to decrease the pH not only is considered as a food safety factor, but also improves glutamic acid production in this bacterium through redirecting metabolic afflux of 2-oxoglutarate towards glutamic acid production and producing ammonia which results in an enhanced glutamic acid production.

**Figure 5:**
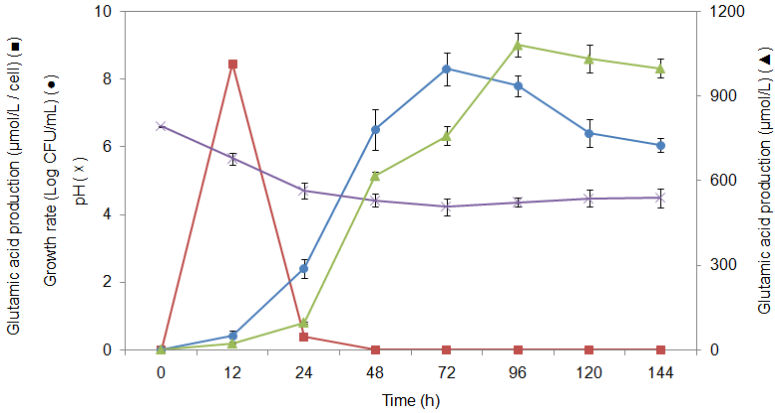
pH, growth rate, glutamic acid production/cell and glutamic acid production profile of *Lactobacillus plantarum* MNZ. Symbols used were: (×), pH profile; (●), growth rate (log CFU/mL); (■), glutamic acid production (μmol/L/cell); (▴), glutamic acid produced (μmol/L).

In order to understand the pH association with glutamic acid production, the pH profile of the fermentation medium was examined and the results presented in Figure 5. It was revealed that between 6 to 72 h of fermentation, the pH immediately decreased from 6.2 to 4.2 followed by a slow increase throughout the remaining fermentation period (Figure 5). Such a reduction in pH value during fermentation might reflect an acid-producing ability of the bacterium, an important feature for the production of quality-fermented foods. The production of organic acids such as lactic and acetic acids is the result of bacterial metabolic activities. The decline in pH is an important characteristic required by the starter strains for acidifying their environment rapidly. The acid production and the accompanying pH decrease are well-known to extend the lag phase of sensitive organisms including food-borne pathogens [33]. Acid production during fermentation, resulting in acidification to pH levels lower than 4.2, constitutes a major food safety factor [34].

*Lactobacillus plantarum* as a facultative hetero-fermentative lactic acid bacterium ferments pentoses through the phosphoketolase pathway and hexoses via glycolysis[18]. In addition, this lactic acid bacterium is capable of mixed acid fermentation which ferments hexoses under specific conditions to various inorganic acids such as acetatic, lactatic and formic acids [35]. The glucose content of the MRS medium can go through the above-mentioned pathways resulting in the production of inorganic acids by the LAB strain MNZ which results in a decrease in pH.

### 2.6. Study of Growth Profile and Association with Glutamic Acid Production

Growth characteristics for LAB strain MNZ were monitored and are presented in Figure 5. The purpose of such a growth characteristic test was to obtain a better understanding about the mechanism of the glutamic acid production in LAB strain MNZ and to find out at which phase of the microbial growth glutamic acid can be produced. Figure 5 shows that *Lactobacillus* cell growth increased exponentially between 18 and 72 h of fermentation in the MRS broth. This stage is recognized as the log phase or exponential phase. It is obvious that the exponential growth phase cannot go on indefinitely. This is due to the fact that the medium is soon depleted of nutrients and enriched with other metabolites. After 72 h of fermentation, a decline in bacterium cell growth was noted and a stationary phase reached. During the stationary phase, the microbial growth rate decreased as a result of nutrient depletion and accumulation of products that may suppress glutamic acid production. This phase is reached as the bacteria begin to exhaust the resources that are available to them.

On the other hand, it was noted that the ratio of glutamic acid production/cell during the first 12 h of fermentation increased to 8.4 μmol/L/cell (Figure 5). Such an elevation of the ratio of glutamic acid production/cell may be associated with the induction of the *gdh* gene. After 12 h of fermentation, the ratio of glutamic acid production/cell drastically decreased to 0.38, 1.9 × 10^−4^, 3.8 × 10^−5^, 1.7 × 10^−5^, 4.1 × 10^−4^ and 8.9 × 10^−4^ μmol/L/cell for 24, 48, 72, 96, 120 and 144 h of fermentation time, respectively. Total glutamic acid production increased between 24 and 96 h although the ratio of glutamic acid production/cell decreased. The increase in glutamic acid production especially after 48 h was due to increasing numbers of bacterial cells in the log phase, which caused an elevation in the glutamic acid yield. The metabolic activity of the bacteria that produced acids resulted in a fall of pH especially for the first 24 h of fermentation. An acidic condition, therefore, could be responsible for triggering the *gdh* gene resulting in elevated glutamic acid production/cell ratio. In this study, a decrease in pH of the fermentation medium was found to be associated with cell growth owing to the glutamic acid production, which was elevated during the log phase of the bacterial growth profile. A similar result was also found in a study by Nakamura *et al*. [21] using *Corynebacterium glutamicum* for glutamic acid production. However, other studies showed that despite the fact that bacterial cells grew well in the synthetic medium, glutamic acid production was depressed during the log phase and only started to increase during the stationary phase [36]. In contrast to our results, it was suggested that glutamic acid production was not associated with growth. Bacterial strain differences and various media containing other factors might be responsible for the differences in the obtained results.

### 2.7. Study of Glutamic Acid-Production Profile

**Figure 6:**
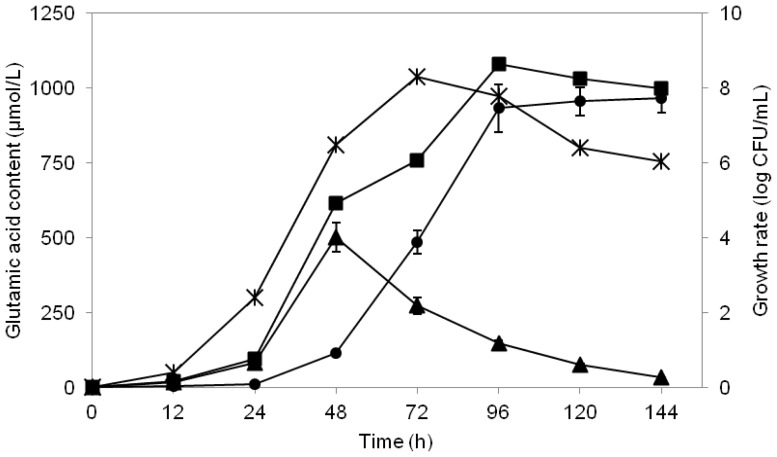
Changes in extra-cellular and intra-cellular glutamic acid content produced by *Lactobacillus plantarum* MNZ cultured in MRS medium supplemented with 12% glucose at 30 °C. Symbols used were: (▴) Intra-cellular glutamic acid; (●) extra-cellular glutamic acid; (■) total glutamic acid; (×) growth profile. Values are mean ± standard deviation of three independent experiments.

The glutamic acid-producing ability of the isolate MNZ was assessed by conducting a time course analysis of intra-cellular and extra-cellular glutamic acid contents in this strain in a culture medium (MRS broth supplemented with 12% glucose). The results are shown in Figure 6. The highest intra-cellular glutamic acid content (502 μmol/L) was achieved after 48 h of cultivation. While the highest extra-cellular glutamic acid biosynthesis, reaching 933 μmol/L, was attained after 96 h of cultivation. Total glutamic acid production (intra- and extra-cellular glutamic acid) was found to be at a maximum (1082 μmol/L) at 96 h of cultivation.

Production of glutamic acid in bacteria is mostly dependent on the cytoplasmic glutamate dehydrogenase (GDH) which catalyzes this amino acid formation from α-ketoglutarate [37]. This enzyme has been demonstrated to occur in high frequency and activity in *Lactobacillus plantarum* with the GDH-encoding gene (*gdh*) implying such an elevated activity [27]. On the other hand, the intra-cellular concentration of glutamic acid in this study regularly decreased after 48 h to such an extent—owing to its secretion into the extra-cellular medium—that the feedback inhibition which regulates the internal glutamic acid pool is abolished [38]. Recently, a glutamic acid exporter in a bacterium was found responsible for excreting glutamic acid to the extra-cellular medium [21].

The mutual effect of a feedback inhibition system in the bacterium along with the glutamic acid permease caused the decline of the intra-cellular glutamic acid in *Lactobacillus plantarum* after 48 h of fermentation process. This research finding along with the reported localization of glutamic acid dehydrogenase in cytoplasm suggests that glutamic acid was synthesized in the cytoplasm of *Lactobacillus plantarum* and then secreted into the culture medium.

To gain a better insight of the mechanism by which glutamic acid is secreted into the extra-cellular medium, three models have been described over the past several years as the crucial steps for glutamic acid efflux. These included functional inversion of uptake systems, diffusion and the existence of particular excretion systems [30]. Recently it was suggested that the mechanism of glutamic acid production in bacteria is not mostly related to the cell membrane structure, but that the production of this amino acid is caused by an alteration in metabolic efflux which leads to glutamic acid biosynthesis [31]. Furthermore, two genes in bacteria were shown to be involved in the glutamic acid efflux properties [39] although the functions of these genes still remain unclear [40]. Consequently, appropriate investigations are required to discover the mechanisms involved in glutamic acid production in Lacobacilli.

## 3. Experimental Section

### 3.1. Samples

Six locally available fermented foods including *tempoyak* (fermented durian flesh), *tempeh* (fermented soybean), *tapai ubi* (fermented tapioca), *tapai pulut* (fermented glutinous rice), *budu* (fish sauce) and *cincalok* (fermented shrimp sauce) were purchased from wet markets in Perlis, Kelantan and Selangor states, peninsular Malaysia as LAB-strain local sources.

### 3.2. Chemicals and Media

HPLC-grade solvents were purchased from Sigma (Sigma-Aldrich, St. Louis, MO, USA). HPLC-grade water was prepared using a Sartorius apparatus (Arium 611 UV, Sartorius Stedim Biotech, Göttingen, Germany). MRS medium was purchased from Difco (Detroit, MI, USA).

### 3.3. Isolation of Lactic Acid Bacteria

Isolation of lactic acid bacteria (LAB) from fermented food products was performed according to the method described by Adnan and Tan [41] with minor modifications. Each sample (10 g) was separately blended with 90 mL of 0.85% NaCl solution for 2 min (Lab Blender Seward, Stomacher 400). This blended food (10 mL) was mixed with MRS broth (90 mL) in a 250 mL Erlenmeyer flask. The broths containing the food samples were enriched with glucose (2% *w*/*v*). The flasks were incubated at 30 °C, 100 rpm, for 7 days. Aliquots of the culture from each of the flasks were serially diluted from 10_1_ to 10_12_ times and 0.1 mL of each dilution was spread evenly on MRS agar plates. Colonies of LAB were counted on MRS agar plates after anaerobic incubation for 72 h at 30 °C in GasPaks jars (GasPaks System, BBL) and colonies were reported as log_10_ CFU/mL. Colonies with distinct morphological differences such as color, size and shape were randomly picked from countable MRS agar plates and subcultured on fresh MRS agar plates. Pure colonies were maintained in 20% *v*/*v* glycerol in MRS broth for storage at −80 °C. Each of the isolates was first tested for catalase reaction based on bubble formation after applying 3% hydrogen peroxide solution on the cells. The isolates with catalase-negative results were Gram-stained, and those with Gram-positive activity were considered as lactic acid bacteria for further experiments and analyses.

### 3.4. Quantification of Glutamic Acid-Producing LAB

LAB isolates obtained from various fermented foods were assessed for their potential to produce glutamic acid in a basal medium. The LAB isolates were grown in MRS broth in 15 mL test tubes with yellow caps at 30 °C for 7 days under anaerobic condition (GasPaks System, BBL). After anaerobic incubation, each test tube was quantified for extra-cellular glutamic acid and intra-cellular glutamic acid (inside the bacterial cells).

### 3.5. Extraction of Glutamic Acid in MRS Broth

The contents of extra-cellular glutamic acid accumulated in the culture medium were extracted according to the method of Yang [42] with minor modifications. First, the culture broth was separated from cells by centrifugation (8000× *g* for 15 min at 4 °C) and the supernatant was diluted 50-fold with 7% (*v*/*v*) of glacial acetic acid. The diluted sample was centrifuged at 8000× *g* for 15 min at 4 °C, and the supernatant was filtered using Nylon 0.22-μm-pore-size membrane and then collected for further analysis.

Intra-cellular glutamic acid was determined following the method described by Komatsuzaki [43] with minor modifications. First, the cells cultured in MRS broth at 30 °C for 7 days, were separated from the culture broth by centrifugation (8000× *g* for 15 min at 4 °C); the cells were washed with 0.9% NaCl three times, and re-suspended in 20 mL of phosphate buffer saline (PBS, pH 7.0) which consisted of 8.0 g NaCl, 0.2 g KCl, 0.91 g Na_2_HPO_4_, 0.12 g KH_2_PO_4_. The cells were suspended in 1.0 mL of 75% (*v*/*v*) ethanol, the homogenate was centrifuged at 8000× *g* for 15 min at 4 °C, and the supernatant was filtered using a Nylon 0.22 μm/L pore-size filter. A 100 mL proportion of the filtered supernatant was collected for quantitative analysis of glutamic acid concentration.

### 3.6. Quantitative Analysis of Glutamic Acid

Glutamic acid extracted from MRS broth was subjected to derivatization according to the method described by Rossetti and Lombard [44]. Quantitative measurement of the glutamic acid was performed by running an HPLC analysis of the glutamic acid according to the method described by Yang [42]. The derivatized samples were dissolved in 200 mL of initial mobile phase, consisting of a mixture of 60% solution A (aqueous solution of 10.254 g sodium acetate, 0.5 mL tri ethylamine and 0.7 mL acetic acid in 1000 mL, final pH 5.8), 12% solution B (acetonitrile) and 28% solution C (deionized water). Gradient HPLC separation was performed on a Shimadzu (Kyoto, Japan) LC 20AT apparatus, consisting of a pump system, a CTO-10ASVP model oven with a 20 μL injection loop injector, and a model SPD-M20A PDA (Photo Diode Array) Detector, in conjunction with a DELL Optiplex integrator. A Prevail C18 column (250 mm × 4.6 mm I.D., particle size 5 μm/L, Alltech, IL, USA) was used during the analysis. The mobile phase for the gradient elution was pumped at 0.6 mL/min flow rate and 27 °C temperature, and glutamic acid detection was performed at 254 nm.

### 3.7. Identification of LAB Isolates

#### 3.7.1. Phenotypic Identification

Microscopic and conventional biochemical and physiological techniques were used to initially characterize all the LAB isolates. The isolates were individually propagated in MRS broth. Each strain was primarily subjected to catalase and Gram-reaction tests. Based upon the phenotypic characteristics, only the catalase-negative and Gram-positive isolates were selected. The selected LAB isolates were also tested for colony formation, cell morphologies, and cell grouping using a light microscope (NIKON YS 100). Further identification of the superior glutamic-acid-producing isolate was carried out by employing API 50 CHL fermentation strips (BioMérieux, Marcy l’Etoile, France) at 30 °C according to the manufacturer’s instructions.

#### 3.7.2. Genotypic Identification

The LAB strain with superior glutamic acid production was identified by 16S rRNA gene sequencing analysis. Genomic DNA from the LAB selected was extracted according to the method described by Sambrook [45]. The 16S rRNA gene amplification was performed using a genomic DNA sample as template and following universal primer pairs; 27F (5′ AGAGTTTGATCCTGGCTCAG 3′) and 1492R (5′ GTTTACCTTGTTACGACTT 3′). The following thermal cycle was used: 95 °C for 3 min; 40 cycles of: 95 °C for 30 s (denaturation), 55 °C for 55 s (annealing), and 72 °C for 60 s (extension); and one cycle final primer extension 72 °C for 10 min. The purified PCR products using QIAquick Gel Extraction Kit (QIAGEN, Germany) were sequenced by First BASE Laboratories Sdn. Bhd. (Shah Alam, Selangor, Malaysia). The 16S rRNA sequences were aligned and compared with other 16S rRNA genes in the GenBank by using the NCBI Basic Local Alignment Search Tools, nucleotide (BLASTn) program [46]. The 16S rRNA gene sequence described in this study was deposited into the Genbank Data Library and assigned the accession number HM175883.

## 4. Conclusion

A new glutamic acid-producing strain of Lactobacilli isolated from a traditional fermented food locally available in Malaysia was identified as *Lactobacillus plantarum* MNZ according to the phenotypic and genotypic tests. Evaluation of the intra- and extra-cellular glutamic acid content confirmed that this amino acid was produced inside the cell and excreted into the extra-cellular medium. Among various carbon sources tested, the finding showed that glucose not only supported *Lactobacillus plantarum* growth, but also significantly enhanced glutamic acid production by this LAB strain. Studying the influence of other factors showed that a 7% (*w*/*v*) concentration of ammonium nitrate and a pH 4.5 improved glutamic acid production. The physiological characteristics of *Lactobacillus plantarum* including pH profile as investigated in this study support its potential use as starter culture in fermented foods while improving glutamic acid production as well. It is essential to select LAB strains with a suitable acid production profile to develop fermented foods with optimum levels of glutamic acid as a precursor of GABA. This new glutamic acid-producing LAB strain could be utilized for mass production of GABA-rich products, thus accelerating the development of functional fermented foods to benefit the consumers.

## Figures and Tables

**Table 1:** Characteristics of the LAB isolates which biosynthesized glutamic acid in MRS broth.

Type of Sample	Total LAB isolates	Total LAB with glutamic acid production	Glutamic acid production range (μmol/L)
Fermented soybean	53	14	20–489
Fermented durian flesh	42	5	3.2–20
Fermented tapioca	21	3	34–59
Fermented glutinous rice	26	3	18–65
Fermented shrimp sauce	27	4	2–11
Fermented fish sauce	49	6	22–106
